# Editorial Note: Machine and deep learning algorithms for sentiment analysis during COVID-19: A vision to create fake news resistant society

**DOI:** 10.1371/journal.pone.0357518

**Published:** 2026-09-03

**Authors:** The PLOS One Editors

The *PLOS One* Editors issue this Editorial Note to inform readers that the articles cited in this article [1] as References 13, 17, and 71 do not appear to support the corresponding cited statements.
